# Human Cryptic Host Defence Peptide GVF27 Exhibits Anti-Infective Properties against Biofilm Forming Members of the *Burkholderia cepacia* Complex

**DOI:** 10.3390/ph15020260

**Published:** 2022-02-21

**Authors:** Andrea Bosso, Rosa Gaglione, Rocco Di Girolamo, Edwin J. A. Veldhuizen, Pilar García-Vello, Salvatore Fusco, Valeria Cafaro, Maria Monticelli, Rosanna Culurciello, Eugenio Notomista, Angela Arciello, Elio Pizzo

**Affiliations:** 1 Department of Biology, University of Naples Federico II, 80126 Naples, Italy; andrea.bosso@unina.it (A.B.); valeria.cafaro@unina.it (V.C.); maria.monticelli@unina.it (M.M.); rosanna.culurciello@unina.it (R.C.); notomist@unina.it (E.N.); 2 Institute of Biochemistry and Cellular Biology—National Research Council (CNR), Via Pietro Castellino 111, 80131 Naples, Italy; 3 Department of Chemical Sciences, University of Naples Federico II, 80126 Naples, Italy; rosa.gaglione@unina.it (R.G.); rocco.digirolamo@unina.it (R.D.G.); pilar.garciadelvellomoreno@unina.it (P.G.-V.); anarciel@unina.it (A.A.); 4 Istituto Nazionale di Biostrutture e Biosistemi (INBB), 00136 Rome, Italy; 5 Section Immunology, Division Infectious Diseases & Immunology, Department of Biomolecular Health Sciences, Faculty of Veterinary Medicine, Utrecht University, 3584 CL Utrecht, The Netherlands; e.j.a.veldhuizen@uu.nl; 6 Department of Biotechnology, University of Verona, 37134 Verona, Italy; salvatore.fusco@univr.it; 7 Centro Servizi Metrologici e Tecnologici Avanzati (CeSMA), Complesso Universitario di Monte Sant’Angelo, Via Cinthia 21, 80126 Naples, Italy

**Keywords:** host defence peptides, antimicrobial peptides, anti-biofilm agents, LPS neutralisation, agglutinating activity, immunomodulatory activities, synergy

## Abstract

Therapeutic solutions to counter *Burkholderia cepacia* complex (Bcc) bacteria are challenging due to their intrinsically high level of antibiotic resistance. Bcc organisms display a variety of potential virulence factors, have a distinct lipopolysaccharide naturally implicated in antimicrobial resistance. and are able to form biofilms, which may further protect them from both host defence peptides (HDPs) and antibiotics. Here, we report the promising anti-biofilm and immunomodulatory activities of human HDP GVF27 on two of the most clinically relevant Bcc members, *Burkholderia multivorans* and *Burkholderia cenocepacia*. The effects of synthetic and labelled GVF27 were tested on *B. cenocepacia* and *B. multivorans* biofilms, at three different stages of formation, by confocal laser scanning microscopy (CLSM). Assays on bacterial cultures and on human monocytes challenged with *B. cenocepacia* LPS were also performed. GVF27 exerts, at different stages of formation, anti-biofilm effects towards both Bcc strains, a significant propensity to function in combination with ciprofloxacin, a relevant affinity for LPSs isolated from *B. cenocepacia* as well as a good propensity to mitigate the release of pro-inflammatory cytokines in human cells pre-treated with the same endotoxin. Overall, all these findings contribute to the elucidation of the main features that a good therapeutic agent directed against these extremely leathery biofilm-forming bacteria should possess.

## 1. Introduction

Multi-drug resistance in bacteria is one of the most pressing global health issues of our time [1,2]. Current approaches to counteract antimicrobial resistance focus primarily on two main challenges, i.e., ensuring a more prudent and efficient use of existing antibiotics and making available new antimicrobial agents. The latter clearly requires great efforts and significant investments addressed to the identification and development of new effective molecules [3]. An extremely promising source of new anti-infective molecules is represented by host defence peptides (HDPs), short molecules (10–50 residues in length) mostly characterised by a net positive charge and a high proportion of hydrophobic amino acids, naturally occurring and produced by most living beings, spanning from microbes to mammals [4,5]. HDPs are a widely distributed family of molecules for the treatment of various bacterial infections, including opportunistic ones, with additional properties concerning the ability to steer innate and adaptive immunity [4,6]. HDPs attenuate immune response through several types of actions, as binding to endotoxins, contribution to the enhancement of innate immune processes by promoting chemotaxis of neutrophils, monocytes, eosinophils, dendritic cells, and T-lymphocytes to the site of infection, stimulation of chemokine production, and modulation of wound healing/re-epithelialisation of injured infected tissues [7,8,9,10,11]. This wide range of properties attribute to HDPs a critical role in mammalian innate immunity and makes them very attractive as an effective alternative to conventional antibiotics for developing new anti-infective agents [12,13]. However, resistant bacteria intrinsically able to counteract HDPs activity have been reported, including the well characterised group of *Burkholderia cepacia* complex (Bcc). This group of Gram negative closely-related species phylogenetically but not phenotypically distinguishable, known also as genomovars, cover a prominent role in determining serious health risks with drastically limited treatment options in immunocompromised individuals [14,15]. Bcc bacteria, particularly *B. cenocepacia* and *B. multivorans* strains, generally display an intrinsic resistance to clinically relevant antibiotics [14,16] mediated by mechanisms as enzymatic inactivation (β-lactamases, aminoglycoside-inactivating enzymes, and dihydrofolate reductase), alteration of drug targets, cell wall impermeability, and activation of efflux pumps [17,18]. Their pathogenicity is also promoted by several virulence determinants [16,19,20] that, together with an extraordinary metabolic versatility [19] and a strong propensity to form biofilm, allow their adaptation to a wide range of environments, thus making the treatment of *Burkholderia* infections particularly difficult. Because of its clinical importance, the biofilm of Bcc is thoroughly investigated. In recent years, alongside the identification of new extracellular polymeric substances (polysaccharides) [20], the detection of several proteins transported by outer membrane vesicles has led to the consideration that these structures are crucial for biofilm physiology [21]. Other proteins, such as extracellular lipases, metalloproteases, and serine proteases are key elements in the interaction of Bcc with host cells [22]. While metalloproteases and serine proteases seem to play a role in the proteolysis of the extracellular matrix and are produced by many but not all the Bcc species [23], lipases are thought to be involved in the invasion, and their production is broadly distributed among the members of the Bcc [24]. Moreover, bacterial surface structures, such as lipopolysaccharides (LPSs), flagella, and pili, are pivotal in the interaction with the host as they are able to subvert innate immune receptors (e.g., toll-like receptors—TLRs), thus inducing a disproportionate pro-inflammatory cytokine response [25]. In particular, LPSs purified from Bcc were found to exhibit in vitro a higher endotoxic activity and to induce a more pronounced cytokine response in human leukocytes and macrophages compared to LPS from several cystic fibrosis clinical isolates of *Pseudomonas aeruginosa* [26,27]. Structural analyses on different LPSs purified from Bcc suggest also that their unusually high pro-inflammatory activity is mostly linked to differences in the acylation state of lipid A [28,29,30]. In addition, the modification of phosphate groups in lipid A with 4-amino-4-deoxy-l-arabinose (l-Ara4N), is a strategy that allows a proper assembly of LPSs at the outer membrane and probably is the most critical determinant for the intrinsic resistance of most of these bacteria to antimicrobial peptides [28]. The picture that emerges from all these considerations clearly indicates that Bcc members evade host innate immune system through multiple mechanisms and, for this reason, the discovery of molecules capable of effectively fighting these pathogens acquires an indisputable priority in the context of multi-drug resistance. Numerous human proteins, not necessarily directly involved in host defence, are potential reservoir of HDPs hidden inside their sequences [29,30,31]. Among these, the 11-hydroxysteroid dehydrogenase-1 β-like (accession number UniProtKB: Q7Z5J1) has proven to be a promising precursor protein. This latter is an enzyme in which a potent antimicrobial peptide, GVF27, was identified and described for its structural and anti-infective properties [32]. This paper focuses on a panel of bioactivities of GVF27 specifically directed against two extremely virulent members of Bcc, such as *B*. *cenocepacia* and *B*. *multivorans*. In particular, we report that GVF27 exerts (i) high anti-biofilm effects towards these two Bcc strains, (ii) the ability to function in combination with the ciprofloxacin antibiotic, (iii) a strong affinity for LPS, and (iv) a good propensity to mitigate the release of pro-inflammatory cytokines in human cells treated with the same endotoxin. Overall, these findings contribute to the elucidation of the main features that a good therapeutic agent directed against these extremely leathery biofilm-forming bacteria should possess.

## 2. Results

### 2.1. Anti-Biofilm Activity of GVF27 on Bcc Clinical Isolates

**Figure 1:**
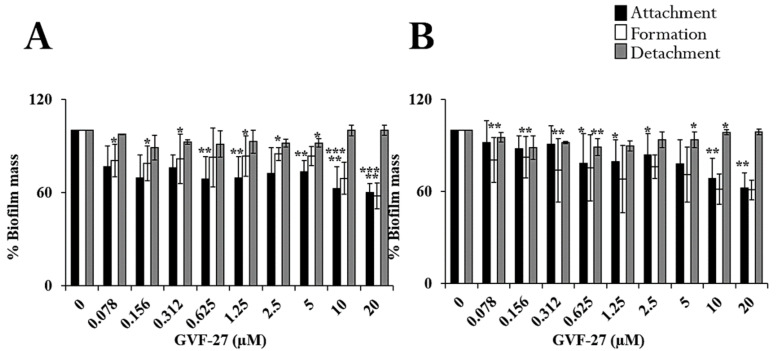
Anti-biofilm activity of GVF27 on *B. cenocepacia* LMG 18863 (**A**) and *B. multivorans* LMG 17582 (**B**) strains. The effects of increasing concentrations of peptide were evaluated either on biofilm attachment (black bars), biofilm formation (white bars), or on pre-formed biofilm (grey bars). Biofilm was stained with crystal violet and measured at 600 nm. Data represent the mean (±standard deviation, SD) of at least three independent experiments, each one carried out with triplicate determinations. * *p* < 0.05, ** *p* < 0.01, or *** *p* < 0.001 were obtained for control versus treated samples.

**Figure 2:**
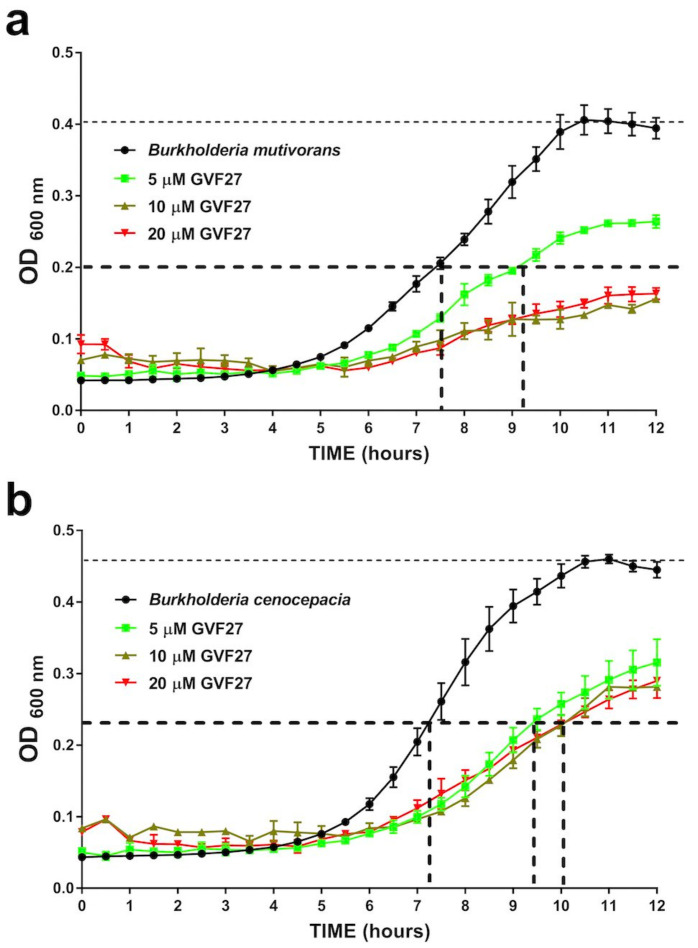
Time-killing curves obtained for GVF27 tested on *B. multivorans* LMG 17582 (**a**) and *B. cenocepacia* LMG 18863 (**b**). Three doubling dilutions are plotted; the highest concentration (red line) corresponds to 2 × MIC of *B. multivorans* LMG 17582 and to 0.5 × MIC of *B. cenocepacia* LMG 18863, respectively. GVF27 was added at time point 0 and monitored until 12 h. Bacterial growth delay was defined as the delay in hours for peptide-treated bacteria to reach half of the maximum OD of the untreated control (OD ½). GVF27 (5 μM) delay of 1.45 h the growth of *B. multivorans* and of 2.5 h the growth of *B. cenocepacia*. At higher doses (10 and 20 μM), GVF27 prevents reaching the OD ½ of *B. multivorans* while, at the same doses, it is not capable to further delay the growth of *B. cenocepacia*.

As previously reported [32], GVF27 proved to exert a powerful antimicrobial activity on biofilm-forming *Escherichia coli*, showing a significant propensity to reduce the microbial load and disrupt the biofilm mass. Here, we evaluated the anti-biofilm properties of GVF27 on Bcc bacteria and, to do this, we selected two clinical isolates: *B. cenocepacia* LMG 18863 and *B. multivorans* LMG 17582. To assess the effects of GVF27 on the three main stages of biofilm development (attachment, formation, and detachment), increasing concentrations of the peptide (from 0.078 to 20 μM) were tested. In each case, following incubation with the peptide, biofilm was analyzed by staining with crystal violet, a dye that stains both cells and extracellular material, thus allowing the determination of the total biofilm mass. As shown in Figure 1, GVF27 was found to be effective at peptide concentrations lower than those required to directly kill planktonic cells (Table 1) or to induce a significant decrease of their growth rate (Figure 2). The latter test indicated that sub-MIC concentrations of GVF27 (5 μM for *B. multivorans* and 5-10-20 μM for *B. cenocepacia*) are enough to affect the growth of planktonic cells; however, even more interesting is the evidence that the same concentrations are effective in inhibiting biofilm adhesion and formation.

**Figure 3:**
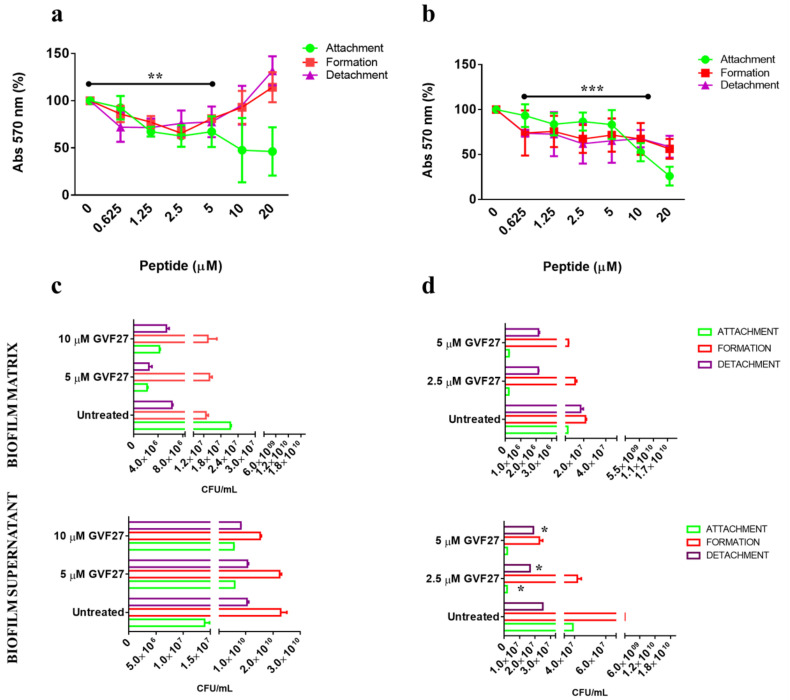
Analysis of *B. cenocepacia* (**a**) and *B. multivorans* (**b**) metabolic activity within the biofilm at different stages of development: attachment (green circle), formation (red square), and detachment (purple triangle). Analysis of CFU within biofilm matrix and supernatant for *B. cenocepacia* (**c**) and *B. multivorans* (**d**) at different stages of development: attachment (green), formation (red), and detachment (purple). Data represent the mean (±standard deviation, SD) of at least three independent experiments, each one carried out with triplicate determinations. * *p* < 0.05, ** *p* < 0.01, or *** *p* < 0.001 were obtained for control versus treated samples.

**Figure 4:**
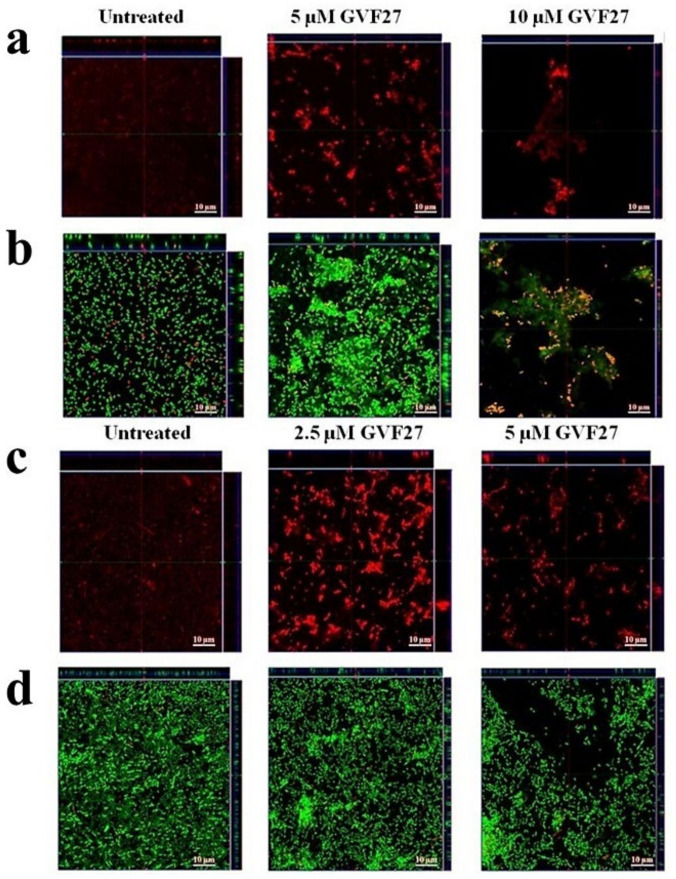
Anti-biofilm activity of GVF27 peptide on the attachment of *B. cenocepacia* LMG 18863 (**a**,**b**) and *B. multivorans* LMG 17582 (**c**,**d**) biofilm. Biofilm cells were stained by using SYPRO™ Ruby (**a**,**c**) and LIVE/DEAD BacLight bacterial viability kit containing Syto-9 (green fluorescence, all cells) and propidium iodide (PI; red fluorescence, dead cells) (**b**,**d**). Scale bar corresponds to 10 μm in all the cases.

**Figure 5:**
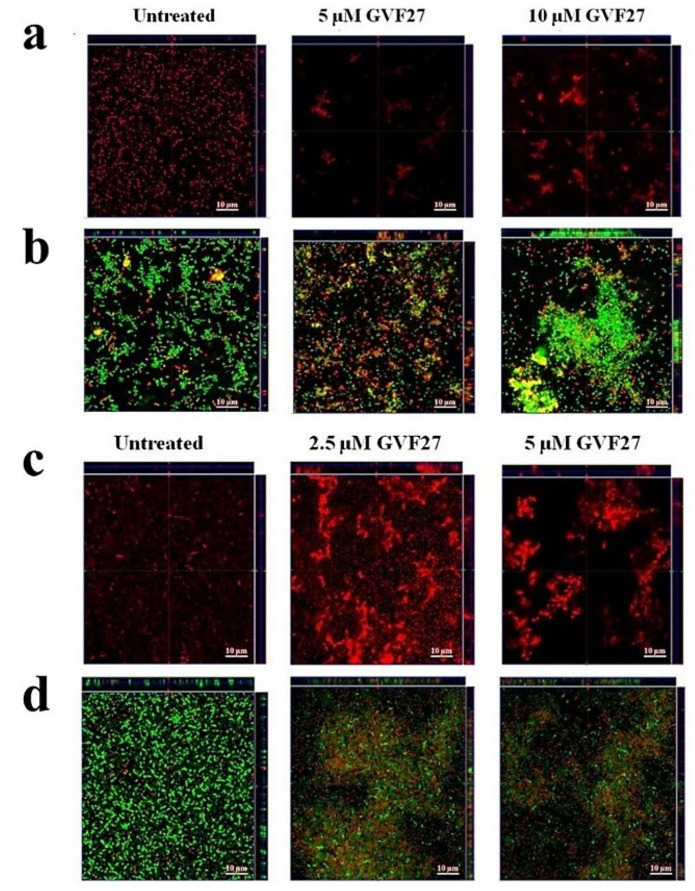
Anti-biofilm activity of GVF27 peptide on the formation of *B. cenocepacia* LMG 18863 (**a**,**b**) and *B. multivorans* LMG 17582 (**c**,**d**) biofilm. Biofilm cells were stained by using SYPRO™ (**a**,**c**) and LIVE/DEAD BacLight bacterial viability kit (**b**,**d**). Scale bar corresponds to 10 μm in all the cases.

**Figure 6:**
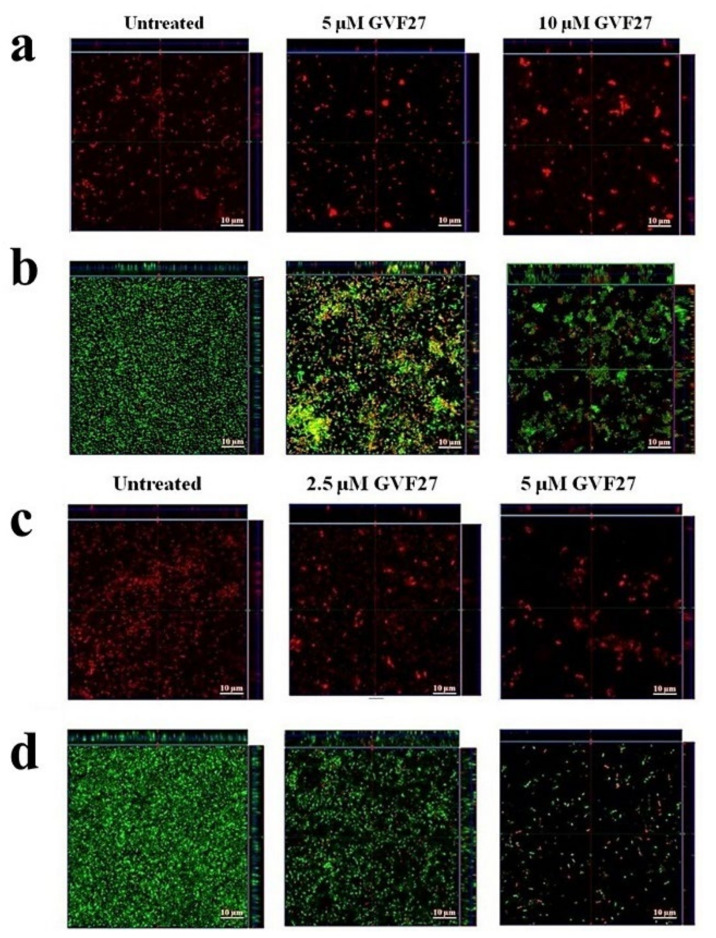
Anti-biofilm activity of GVF27 peptide on preformed biofilm of *B. cenocepacia* LMG 18863 (**a**,**b**) and *B. multivorans* LMG 17582 (**c**,**d**) pre-formed biofilm by CLSM imaging. Biofilm cells were stained by using LIVE/DEAD BacLight bacterial viability kit (**c**,**d**) and SYPRO™ Ruby (**a**,**b**). Scale bar corresponds to 10 μm in all the cases.

Biofilm attachment and formation were affected in a similar percentage in the case of both bacterial strains, whereas apparently GVF27 did not exert significant effects on preformed biofilm even at the highest doses tested. To further investigate anti-biofilm properties of GVF27 on both strains, analyses were also performed by MTT assay (see Figure 3) and CLSM observations (see Figure 4, Figure 5 and Figure 6). MTT assay was carried out to measure bacterial metabolic activity within the biofilm. As shown in Figure 3, this test highlighted a significant dose-dependent efficacy of GVF27 in reducing bacterial viability in the case of both *B. cenocepacia* and *B. multivorans* during the attachment stage. Interestingly, bacterial viability of *B. multivorans* is effectively compromised also during formation and detachment stages, while this behaviour has not been observed in the case of *B. cenocepacia*. In this case, indeed, an apparent increase of metabolic activity is observed during formation and detachment stages. Although statical analyses indicate that this increase is not significant, it has to be highlighted that these data are in agreement with those obtained by counting colonies during the three different phases of biofilm maturation (attachment, formation, and detachment) of both strains (*B. cenocepacia* and *B. multivorans*) after GVF27 administration. Indeed, as evidenced in Figure 3c, a significant decrease of the number of colonies was observed only during biofilm attachment in the case of *B. cenocepacia*. In the case of *B. multivorans*, instead, a significant decrease of colonies number was observed both during attachment and detachment stages (Figure 3d). Furthermore, in order to discriminate and to semi-quantify live cells, dead cells, and extracellular material, we also performed CLSM observations by using LIVE/DEAD BacLight kit and FilmTracer SYPRO Ruby biofilm matrix stain (Figure 4, Figure 5 and Figure 6). The first staining kit exploits two dyes, the green-fluorescent SYTO™ 9 dye and the red-fluorescent propidium iodide, to discriminate between living cells (green) and dead or dying ones with a compromised membrane (red). On the other hand, FilmTracer SYPRO Ruby specifically stains extracellular biofilm matrix proteins.

Images in Figure 4, Figure 5 and Figure 6 clearly indicate that GVF27 is able to affect bacterial viability in a dose-dependent manner by affecting all the three biofilm stages tested. Moreover, images collected in Panels a and c of each figure also suggest that the peptide is able to induce a significant alteration of biofilm matrix architecture.

**Figure 7:**
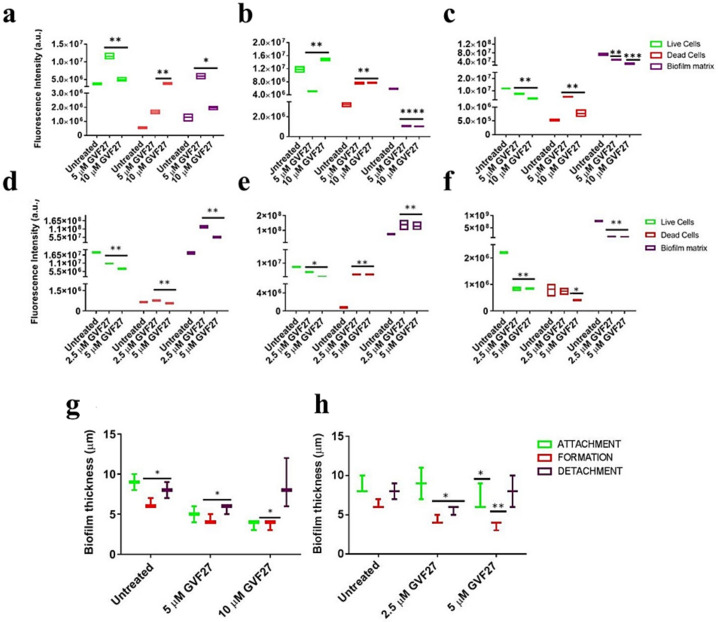
CLSM fluorescence analysis of the effects of GVF27 on *B. cenocepacia* LMG 18863 biofilm ((**a**) attachment, (**b**) formation, and (**c**) detachment) and *B. multivorans* LMG 17582 biofilm ((**d**) attachment, (**e**) formation, and (**f**) detachment). Fluorescence intensity of Syto-9, propidium iodide, and Sypro Ruby Biofilm Matrix Stain were measured by using Zen Lite 2.3 software. 2D reported images are sections of 3D projections of biofilm structure obtained by two independent experiments analyzed per peptide concentration (with triple intra-experimental acquisitions). * *p* < 0.05, ** *p* < 0.01, and *** *p* < 0.001 for treated versus control samples. Analysis of the effects of GVF27 peptide on *B. cenocepacia* LMG 18863 (**g**) and on *B. multivorans* LMG 17582 (**h**) biofilm thickness (green, attachment; red, formation; purple, detachment). 2D reported images are sections of 3D projections of biofilm structure obtained by two independent experiments with triple intra-experimental acquisitions. * *p* < 0.05 and ** *p* < 0.01 for treated versus control samples.

**Figure 8:**
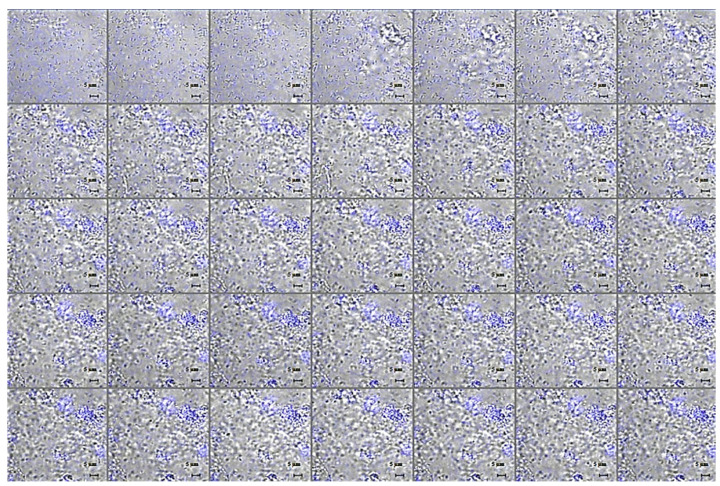
Time course analysis of 5 µM FAM-GVF27 interaction with *B. cenocepacia* LMG 18863 biofilm (0–240 min, 1 acquisition/7.5 min). See Video S1.

This indication is also observable in fluorescence analyses shown in Figure 7a–f, where it appears clearly evident that treatment with the peptide determines a significant decrease of samples fluorescent staining, with effects even more accentuated in the case of *B. cenocepacia* LMG 18863. It is worth mentioning that, even if this observation appears in contrast with evidence collected upon samples staining with crystal violet (Figure 1, grey bars), it is particularly relevant considering that the peptide was tested on three different phases of biofilm development and that, unlike crystal violet staining, analyses by confocal microscopy allow to deeply analyze biofilm morphology, biovolume, and size. This suggests that GFV27 can counteract both the early stages of biofilm matrix organization, during the transition from planktonic to the sessile form, and the advanced ones in which the transition to the sessile form is complete and a peculiar molecular framework has been already formed to protect the cells encapsulated inside. Further analyses were carried out to determine possible effects of GVF27 on total biomass (thickness) of biofilm of both strains. In this regard, once three-dimensional images were collected and optical Z-sections at 1 μm intervals were obtained from the bottom to the top of the biofilm, we determined average thickness measurements by Zen Black software (see Methods) and noted that GVF27 presented a good propensity to mitigate biofilm biomass enhancement of both bacterial strains (Figure 7g,h). As previously shown in Figure 6, GVF27 was able to eradicate preformed biofilm of *B. cenocepacia LMG 18863* (Panel a and b) and *B. multivorans LMG 17582* (Panel c and d) when administered at 5 and 2.5 µM, respectively. However, we can reasonably assume that the peptide was active also at the highest concentrations tested, since the denser thickness detected (purple lines in Figure 6g,h) could be the result of a greater number of eradicated and fluctuating planktonic cells in the scan region (6–12 µm), whereas the maximum coverage surface of the matrix was generally concentrated in the regions between 0 to 6 µm. To support these findings, a time course analysis of the interaction between GVF27 and biofilm components of *B. cenocepacia* LMG 18863 was also performed. To this purpose, the interaction of GVF27, labelled to its *N*-terminus with 5,6-carboxyfluoresceine (FAM-GVF27), with *B. cenocepacia* biofilm has been evaluated over a time interval of 240 min. Time-lapse video of biofilm formation (Video S1) as well as Figure 8 clearly confirm that GVF27 is able to progressively interact with biofilm components and planktonic cells.

### 2.2. Studies of Agglutinating Activity

**Figure 9:**
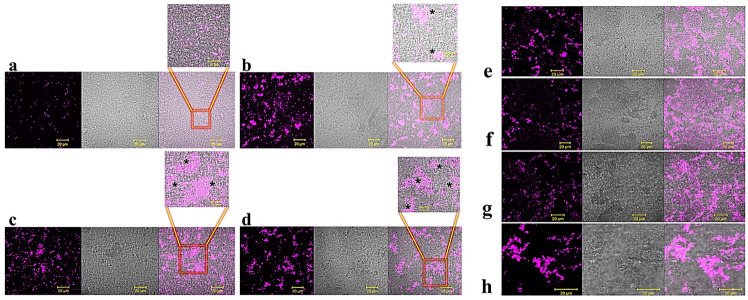
Analysis of GVF27 effects on *B. cenocepacia* LMG 18863 planktonic cells agglutination by CLSM. The biofilm was incubated in the absence (**a**) or in the presence of 0.625 (**b**), 1.25 (**c**), or 2.5 (**d**) µM peptide and stained with wheat germ agglutinin-Oregon Green 488 conjugate. A magnification of each section is shown on the top of the images. Scale bar corresponds to 10 or 20 μm. Analysis of GVF27 effects on *B. cenocepacia* LMG 18863 biofilm agglutination by CLSM. The biofilm was incubated in the presence of 5 (**e**), 10 (**f**), 20 (**g**), or 40 µM peptide (**h**) and stained with wheat germ agglutinin-Oregon Green 488 conjugate. Scale bar corresponds to 20 μm in all the cases.

**Figure 10:**
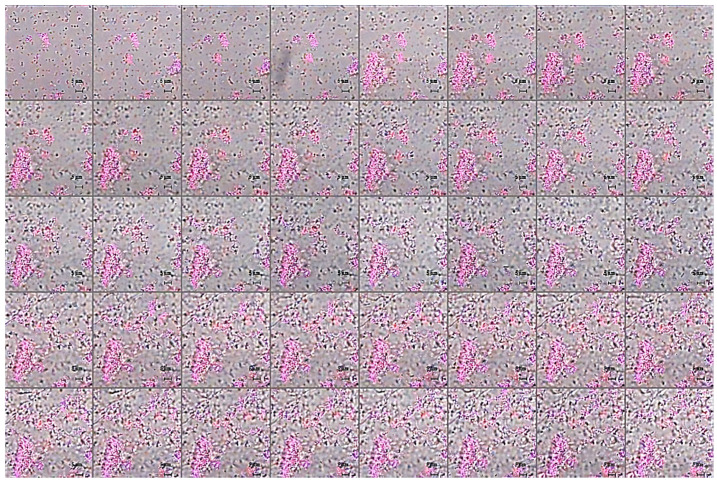
Time course analysis of 5 µM labelled FAM-GVF27 (blue) and FilmTracer™ SYPRO™ Ruby Biofilm Matrix Stain (red) in the presence of *B. cenocepacia* LMG 18863 biofilm (0–240 min, 1 acquisition/7.5 min). See Video S2.

**Figure 11:**
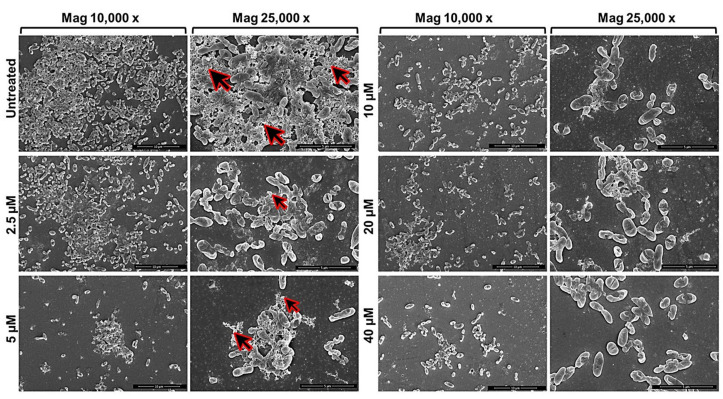
Scanning electron micrographs of biofilm formation (4 h) of *B. cenocepacia* LMG 18863 in the presence of increasing concentrations of GVF27. The magnification scale is shown at the bottom of each micrograph. Black arrows indicate extracellular biofilm matrix.

**Figure 12:**
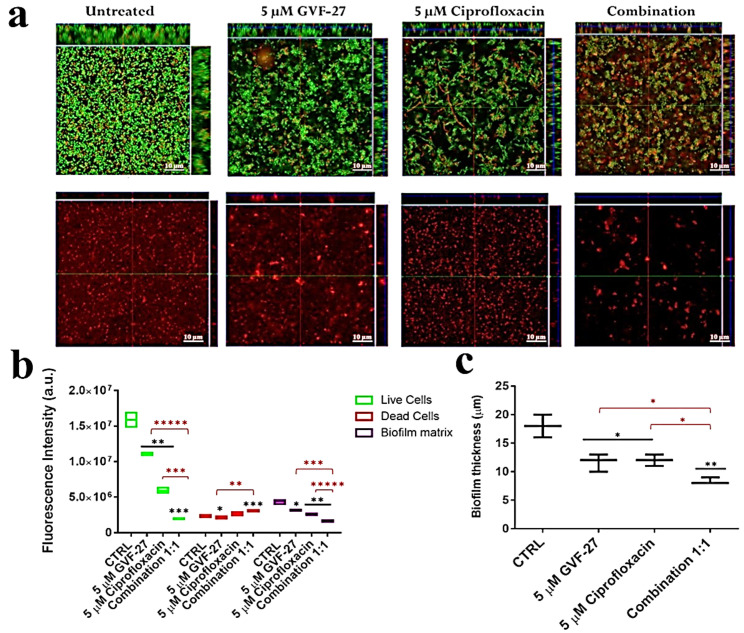
Effects of GVF27, ciprofloxacin, and a combination of the two compounds on preformed biofilm analysed by CLSM. (**a**) Biofilm cells were stained by using LIVE/DEAD BacLight bacterial viability kit. 2D reported images are sections of 3D projections of biofilm structure obtained by laser scanning confocal z-stack using Zen Lite 2.3 software. All the images were taken under identical conditions. Significant differences were indicated as * *p* < 0.05, ** *p* < 0.01, and *** *p* < 0.001 for treated versus control samples (**b**); significant differences were indicated as * *p* < 0.05, ** *p* < 0.01, *** *p* < 0.001, and ***** *p* < 0.00001 for the sample treated with the single agents versus the sample treated with the combination of both agents (**b**,**c**).

In order to protect the bacteria against desiccation and host immune defences, a variety of exopolysaccharides are required for the development and integrity of bacterial biofilm architecture. The production of poly-β1,6-N-acetylglucosamine (PNAG), also known as “polysaccharide intercellular adhesin” (PIA), an important virulence factor well-characterised in different bacterial biofilms, has been documented in several Bcc biofilms [33] in which it contributes to resistance to multiple antibiotics and persistence during chronic infections. Based on previously reported ability of GVF27 to induce LPS aggregation [32], a feature suggesting a putative ability of the peptide to promote bacterial agglutination, a further test was performed on *B. cenocepacia LMG 18863* static biofilms by CLSM upon treatment with GVF27 and staining of PNAG (see methods). As shown in Figure 9, GVF27 was able to promote the agglutination of bacteria during biofilm formation at significantly lower concentration values than MIC values determined on planktonic cells (Table 1). In particular, the minimal agglutination concentration (MAC) value, defined as the minimal peptide concentration able to induce agglutination of bacteria, was estimated to be about 60 times lower than MIC determined on planktonic cells (0.625 vs. 40 μM). This result suggests a very high affinity of GVF27 for specific biofilm components and swimming bacteria. Moreover, as reported in the same figure (Panels e–h), the agglutination induced by GVF27 occurs in a dose dependent manner with a steady state reached between 5 and 40 µM (Panels a–d). To further investigate the agglutinating ability of GVF27, a time-lapse video of the biofilm formation was recorded by using FAM-GVF27 and SYPRO Ruby to stain the extracellular matrix. As visible in the Video S2 as well as in the Figure 10, this peptide is able to progressively interact with biofilm components in a time dependent manner, as highlighted by an overlap between the blue signal (peptide) and the extracellular matrix (red signal), resulting in an accumulation of magenta signal. Possible morphological modifications of *B. cenocepacia LMG 18863* biofilm upon treatment with GVF27 were also analysed by scanning electron microscopy (SEM). As shown in Figure 11 (top left panels), untreated bacteria presented smooth and intact surfaces and appeared embedded into an extracellular biofilm matrix (black arrows). When bacterial biofilm was treated with increasing doses of peptide; instead, a significant decrease or disappearance of biofilm matrix was clearly measurable with a concomitant decrease of cell density. Interestingly, this trend was dependent from peptide dose until 5 µM, after which a kind of plateau was reached likely due to excess of peptide. As Bcc biofilms are commonly recalcitrant to antibiotics, it was also verified whether GVF27 had the ability to synergistically act in combination with antimicrobial agents, which were ineffective as such on these strains. To this purpose, CLSM analyses were performed to evaluate the effects of combinations of GVF27 and ciprofloxacin on preformed biofilms of *B. cenocepacia* LMG 18863. As shown in Figure 12a,b, the mixture composed by 5 μM GVF27 and 5 μM ciprofloxacin induced a more effective reduction of biofilm (with an additive effect) together with the concomitant increase of the number of dead cells embedded into the biofilm matrix with respect to the treatment with single agents. This result was further corroborated by the evidence that the mixture GVF27/ciprofloxacin (1:1, mol/mol) had a significant increased ability to reduce the thickness of the preformed biofilm (see Figure 12c).

### 2.3. Interaction Studies of GVF27 with LPS and Immunomodulation Assays on THP-1 Cells

**Figure 13:**
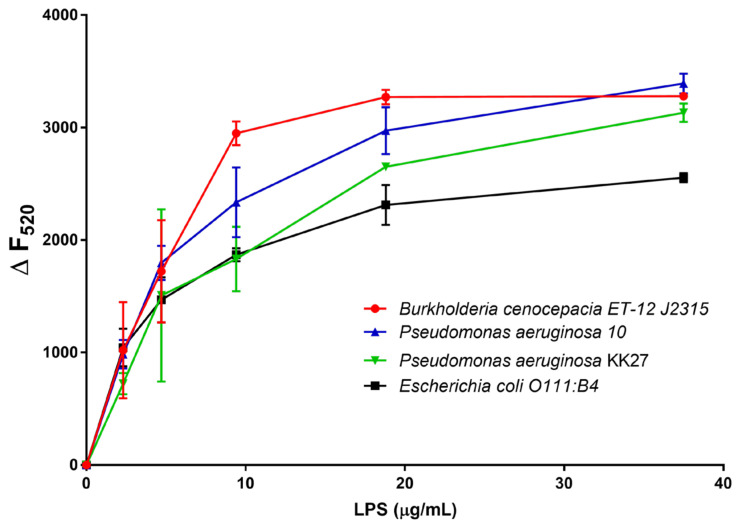
Association determined by analysing fluorescence intensity of FAM-GVF27 in the presence of LPSs isolated from four different bacterial strains. Experiments were performed in 5 mM ammonium acetate buffer (pH 5.0). A total of 1.25 μM FAM-GVF27 was individually titrated with increasing concentrations of LPSs. The fluorescence was monitored at an excitation of 485 nm and at an emission of 520 nm. ΔF_520_ was calculated as follows: (ΔF_520_) = (F_520_ FAM-GVF27_buffer_ − F_520_ FAM-GVF27_LPS_) where F_520_ FAM-GVF27_buffer_ is the fluorescence intensity of FAM-GVF27 measured in buffer and F_520_ FAM-GVF27_LPS_ is the fluorescence intensity of FAM-GVF27 measured in the presence of increasing concentrations of four different LPSs.

LPSs from Bcc are potent immune stimulators that activate human Toll-like Receptor 4 (TLR4) and significantly contribute to host cell damage [34]. Many of the relevant aspects of Bcc behaviour can be ascribed to the peculiar physicochemical properties of the external layer of their cell wall, the architecture of which is in turn determined by the molecular structure of the constituting LPSs. As previously described, the main structural peculiarities of Bcc LPSs are represented by the presence of Ara4N residues on the lipid A backbone and by the Ko substitution in the oligosaccharide core, which together contribute significantly to the neutralisation of anionic charges present on the bacterial cell surface, thus increasing the difficulty of cationic antibiotics (e.g., polymyxin and colistin) to bind to the cell surface. Previous data indicate that GVF27 is able to assume specific conformations in the presence of LPSs isolated from different strains, including also some clinical isolates of *Pseudomonas aeruginosa*, and to promote the formation of larger LPS micelles [32]. These pieces of evidence inspired us to verify whether GVF27 could possess the ability to neutralise LPS associated to Bcc. A first test was carried out using the carboxy-fluorescein labelled version of GVF27 (FAM-GVF27) to analyse its behaviour in the presence of LPSs from *B. cenocepacia* ET-12 J2315, a highly epidemic and virulent strain. Since emission intensity of fluorescein, like that of other high extinction coefficient fluorophores, is very sensitive to self-quenching, it can be expected that binding of fluorescein-labelled peptide to LPS should provoke a decrease in fluorescence intensity. A plot obtained by measuring the fluorescence intensity (at 520 nm) of 1.25 μM FAM-GVF27 emitted in the presence of increasing concentrations of LPS ET-12 J2315 (from 2.4 to 150 μg/mL) is shown in Figure 13. Under the same conditions, the peptide was analysed also in the presence of three different LPSs, two isolated from *P. aeruginosa* strains and one from *E. coli*. Collected results clearly indicate that FAM-GVF27 interacts with LPSs at sub-micellar concentrations.

**Figure 14:**
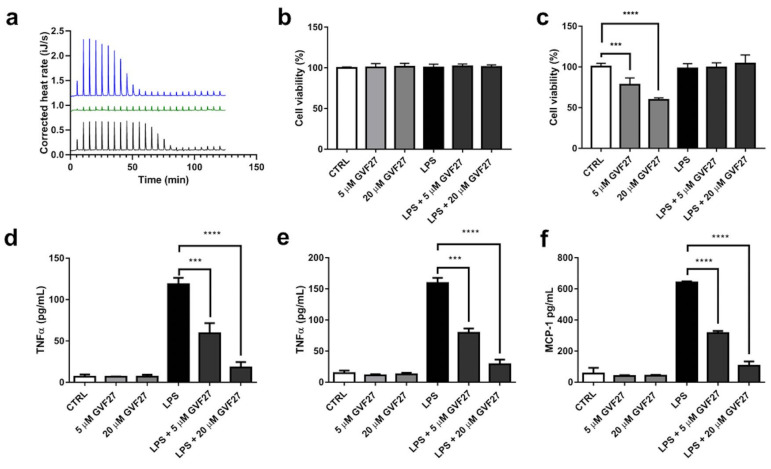
Analyses of GVF27 affinity for LPS ET-12 J2315. (**a**) Representative thermograms of ITC experiments obtained by titration of 200 µM GVF27 into 12.5 µM LPS from *B. cenocepacia* ET-12 J2315 (grey), *E. coli* O111:B4 (blue) or in control HEPES buffer (green). (**b**) Biocompatibility of GVF27 (5 and 20 μM) alone or in the presence of 1 μg/mL LPS ET-12 J2315 on PMA-differentiated THP-1 cells. (**c**) Biocompatibility of GVF27 (5 and 20 μM) alone or in the presence of 1 μg/mL LPS ET-12 J2315 on undifferentiated THP-1 cells. Data presented in Panels b and c were used also to determine GVF27 CC_50_ values, which were found to be equal to 30.49 μM for PMA-differentiated THP-1 cells and >40 μM for undifferentiated THP-1 cells. (**d**) Effects of GVF27 (5 and 20 μM) on the release of TNF-α in untreated or LPS ET-12 J2315 infected undifferentiated THP-1 cells; (**e**) Effects of GVF27 (5 and 20 μM) on the release of TNF-α in untreated or LPS ET-12 J2315 infected PMA-differentiated THP-1 cells. (**f**) Effects of GVF27 (5 and 20 μM) on the release of MCP-1 in untreated or LPS ET-12 J2315 infected PMA-differentiated THP-1 cells. Three independent experiments were performed, and, for all the experimental points, *** *p* < 0.001 or **** *p* < 0.0001 were obtained for control (LPS) versus samples treated with both GVF27 and LPS.

This direct interaction between GVF27 and LPS could be part of a LPS neutralisation mechanism that the peptide exploits to inhibit or strongly counteract the interaction of the endotoxin with LPS-binding protein (LBP) and the subsequent activation of TLR4. To analyse further the possible direct interaction between GVF27 and LPS ET-12 J2315, an isothermal titration calorimetry (ITC) analysis was used, and a strong interaction was observed (Figure 14a). In detail, GVF27 bound to LPS exothermically (ΔH = −28.6 kJ/mol) with a dissociation constant Kd = 61 nM. Interestingly, a positive entropy was measured (ΔS = 48.5 J/mol·K) contributing to binding and indicative of hydrophobic interactions between the peptide and LPS. A strong association was also observed between GVF27 and *E. coli* LPS but with different binding characteristics. The actual dissociation constant was in the same range as for LPS ET-12 J2315 (93 nM), but the enthalpy was twice as high (ΔH = −57.35 kJ/mol), while the entropy was negative (ΔS = −57.4 J/mol·K). This indicates that this interaction is fully enthalpy-driven and, therefore, more dependent on electrostatic interactions between LPS and GVF27. Finally, binding ratios were higher for GVF27 and LPS ET-12 J2315 (almost 2.9 molecules GVF/LPS vs. 1.7), but this is based on estimated molecular weights of both LPSs of 15 kDa, so more detailed studies would be required to determine whether the binding ratio is indeed significantly different. In support of this evidence, further investigations were then carried out to verify whether GVF27 could be also able to reduce TLR4 dependent activation of inflammatory mediators on human THP-1 cells infected with the same endotoxin. Preliminarily to this, biocompatibility of GVF27 on undifferentiated and PMA-differentiated THP-1 cells was verified by testing the effects of the peptide (at 5 and 20 μM), and slightly toxic effects were detected only on undifferentiated THP-1 cells at the highest concentration tested (Figure 14b,c). Interestingly, when GVF27 was tested at the same doses but in the presence of 1 μg/mL LPS ET-12 J2315, undifferentiated THP-1 cell viability was restored to values that were identical to those obtained in the case of untreated cells, thus highlighting once again the ability of the peptide to bind to the endotoxin, thus becoming unavailable to the interaction with cells (Figure 14c). ELISA experiments were then performed to investigate whether GVF27 was able to alter TNF-α and MCP-1 release in LPS treated undifferentiated and PMA-differentiated THP-1 cells. The results shown in Figure 14d,e clearly support the hypothesis that GVF27 is able to interfere with the infection. Indeed, as shown in Figure 14, both in the case of LPS induced undifferentiated and PMA-differentiated THP-1 cells, GVF27 mitigates in a dose-dependent manner the release of TNF-α, and the same promising trend was obtained also for MCP-1 in LPS-induced PMA-differentiated THP-1 cells (Figure 14f).

## 3. Discussion

Epidemiological surveys revealed that the most clinically relevant Bcc species, especially in immunocompromised patients, are *B. multivorans* and *B. cenocepacia* [35]. Treatment of infections caused by these species is often poorly effective due to their inherently resistance to antimicrobial agents [36]. One of the main resistance determinants in these species is represented by the constitutive presence of Ara4N linked to the lipid A phosphate groups in the LPS that eliminates the negative charge required for polymyxin binding and presumably blocks the self-promoted uptake generally utilised by polycationic antimicrobials. Besides this peculiar composition of LPS, the contributions of other genes, such as ispH (involved in the synthesis of isoprenoids), norM (an efflux pump-encoding gene), and hpnJ (encoding hopanoid), as well as enzymatic inactivation and alteration of drug targets, play roles in the multifaceted mechanisms that result in high levels of resistance to polycationic antimicrobials in these Bcc members [36,37]. In addition, biofilm-associated cells increase tolerance to antimicrobials by reduction of drug penetration, a lower growth rate of sessile cells, the expression of specific resistance genes, and the presence of persister cells typically considered as “dormants” [38]. Even when therapies are based on high doses of antibiotics known to be effective against Bcc species in vitro, it is often impossible to clear the infection. Nevertheless, as there are exceptions to every rule and this could also apply to these organisms, here, we report the significant anti-biofilm and immunomodulating properties against *B. multivorans* and *B. cenocepacia* of GVF27, a human cryptic host defence peptide derived from the 11-hydroxysteroid dehydrogenase-1 β-like [32]. Multiple approaches here described clearly indicate that GFV27 can counteract both the early stages of biofilm matrix organization, during the transition from planktonic to the sessile form, and the advanced ones in which the transition to the sessile form is complete and a peculiar molecular framework has been already formed to protect the cells encapsulated inside. According to CLSM, time-dependent localisation and SEM analyses, GVF27 appears to progressively interact with different biofilm components and cells, while inducing a significant reduction and alteration of biomass. Although this behavior does not appear evident by crystal violet biomass determination, it has to be highlighted that data obtained by confocal laser scanning microscopy on the three main phases of biofilm development appear very robust considering that, compared to crystal violet staining, this kind of analysis allows a deep evaluation of biofilm morphology, biovolume and size. It has also to be highlighted that, by CLSM analyses, staining with Sypro Ruby was found to be stronger in the case of treated samples with respect to control ones, thus indicating a deconstruction of biofilm matrix induced by the treatment with the peptide and a consequent higher exposure of proteins, normally embedded in biofilm matrix, that can be efficiently stained by Sypro Ruby. Indeed, it has been extensively reported that Sypro Ruby staining patterns may widely vary depending on the organism and the matrix composition, which might be responsible for different degrees of permeability of biofilm matrix to this dye [39]. Hence, we can affirm that collected data support the hypothesis that GVF27 is endowed with a significant antibiofilm activity. It is also widely reported that antibiotics for therapeutic use (aminoglycosides, fluoroquinolones, tetracycline, and other) work poorly in chronic infections, acting in some cases as inter-microbial signaling agents that stimulate bacterial biofilm formation instead as weapons [40,41]. Interestingly, in our experiments, we found that anti-biofilm properties of GVF27 are even more pronounced when the peptide is administered in combination with ciprofloxacin. Indeed, CLSM analyses performed to evaluate the effects of combinations of GVF27 and ciprofloxacin on preformed biofilms of *B. cenocepacia* LMG 18863 allowed us to detect a more effective reduction of biofilm together with a concomitant increase of the number of dead cells embedded into the biofilm matrix with respect to the treatment with single agents, thus indicating a significant additive effect. These data further support the idea that GVF27 could ideally act as a treadmill for those agents unable to overcome the narrow and impervious scaffolding of the biofilm. Indeed, this opens the way to the development of suitable combinatorial therapeutic approaches that might allow significantly lowering the doses of the antibiotic, with a consequent decrease of the appearance of resistant clinical isolates, an undesired phenomenon strongly affecting conventional antibiotics efficacy. Based on collected data, we hypothesized that the ability of GVF27 to affect biofilm architecture might facilitate ciprofloxacin access to the cells embedded in biofilm matrix, with the consequence that the antibiotic penetrating the biofilm may exert strong bactericidal affects even at concentrations much lower than those normally required. This hypothesis is in agreement with recent data reported for novel antifungal furanone derivatives [42].

Most Bcc isolates recovered from immunocompromised patients produce large amounts of exopolysaccharides (EPS), such as poly-β-1,6-N-acetylglucosamine (PNAG), suggesting a possible role of this EPS in Bcc pathogenesis [43]. To investigate the putative agglutinating activity of GVF27, tests on *B. cenocepacia* static biofilms upon treatment with the peptide and staining of PNAG were also performed. Minimal agglutination concentration (MAC) values detected suggest a very high affinity of GVF27 for PNAG and also for swimming bacteria. In addition, SEM analyses highlighted that the extracellular matrix produced by *B. cenocepacia* significantly reduces in the presence of GVF27. All these findings, in agreement with other studies in which it was highlighted that PNAG is needed to form and maintain the integrity of *Burkholderia* biofilms [33], support the idea that the peptide may exert its antibiofilm activity by mechanisms that go beyond a direct microbicidal effect, thus suggesting, as above mentioned, that their use, alone or in combination with other conventional or unconventional drugs, may represent an effective strategy to target biofilm cells. In addition, interaction studies of GVF27 with LPS isolated from *B. cenocepacia* ET-12 J2315 strain, a highly epidemic and virulent strain, indicate that the peptide is able to efficiently bind also to this endotoxin. This propensity was observed not only in vitro by ITC experiments but also in human monocyte cultures in which it was possible to highlight a significant mitigation of inflammatory mediators when cells were subjected to treatment with the endotoxin. In a previous work, GVF27 was found to be not toxic when tested on murine Raw 264.7 cells and human HaCat and HeLa cells; in this work, GVF27 was tested on PMA-differentiated and undifferentiated THP-1 cells and its toxicity was detected at CC_50_ close to MIC values determined against *B. cenocepacia* (data not shown). Nevertheless, we highlighted that GV27 is both able to inhibit early stages of Bcc biofilm formation and LPS proinflammatory properties at concentrations lower than those necessary to have cytotoxic effects. 

## 4. Materials and Methods

### 4.1. Materials

All chemicals were purchased from Merck KGaA (Darmstadt, Germany), unless specified otherwise.

### 4.2. Peptide Synthesis

GVF27, FAM-GVF27, and LL-37 were synthesised by the solid-phase method (CASLO ApS, Lyngby, Denmark). FAM-GVF27 presents 5,6-carboxyfluoresceine fluorochrome conjugated to the *N*-terminus of peptide. The purity of all the three peptides was determined by analytical high-performance liquid chromatography (HPLC) to be higher than 95%. Trifluoroacetic acid (TFA) was removed, and the molecular weight was confirmed by MALDI-TOF mass spectrometry. Peptide sequences are reported in Table 2.

### 4.3. Bacterial Strains and Growth Conditions

Clinical isolates *B. cenocepacia* LMG 18863 and *B.multivorans* LMG 17582 were kindly provided by Dr. Alessandra Bragonzi (Infection and CF Unit, San Raffaele Scientific Institute, Milan, Italy). Both bacterial strains were grown in Muller Hinton Broth (MHB, Becton Dickinson Difco, Franklin Lakes, NJ, USA) and on Tryptic Soy Agar (TSA; Oxoid Ltd., Hampshire, UK). In all the experiments, bacteria were inoculated and grown overnight in MHB at 37 °C. The next day, bacteria were transferred to a fresh MHB tube and grown to mid-logarithmic phase.

### 4.4. Antimicrobial Activity

The antimicrobial activity of GVF27, FAM-GVF27, and LL-37 on *B. cenocepacia* LMG 18863 and *B. multivorans* LMG 17582 was determined by using the broth microdilution method, as previously described [44]. Bacteria were grown to mid-logarithmic phase in MHB at 37 °C, then diluted to 2 × 10^6^ Colony Forming Units (CFU)/mL in Difco 0.5× Nutrient Broth (NB) (Becton-Dickenson, Franklin Lakes, NJ, USA) and mixed 1:1 *v*/*v* with two-fold serial dilutions of peptides. Following over-night incubation, MIC_100_ values were determined as the lowest peptide concentration responsible for no visible bacterial growth. All the experiments were carried out in three independent replicates.

### 4.5. Cell Culture and Differentiation

Human monocytic cells THP-1 (ATCC, TIB-202) were cultured in Roswell Park Memorial Institute 1640 medium (RPMI) purchased from Lonza (Basel, Switzerland) completed with 10% *v*/*v* fetal bovine serum, 100 μg/mL penicillin, and 100 μg/mL streptomycin and stored in a humidified 37 °C incubator with 5% CO_2_/95% air. Cells were periodically checked at the microscope to ensure normal morphology. THP-1 cells were differentiated using 2 nM phorbol 12-myristate 13-acetate (PMA) for 96 h at 37 °C with 5% CO_2_/95% air.

### 4.6. Cytotoxicity Assays

Cytotoxic effects of GVF27 on undifferentiated or PMA-differentiated THP-1 cells were determined by performing the 3-(4,5-dimethylthiazol-2-yl)-2,5 diphenyltetrazolium bromide reduction inhibition assay (MTT assay), designed to be used for the spectrophotometric quantification of cell proliferation. Briefly, 2 × 10^4^ cells were seeded into a 96-well plate (round-bottomed for undifferentiated suspended cells and flat-bottomed for differentiated adherent cells) and incubated at 37 °C in the presence of 5% CO_2_. Medium was then replaced with 100 μL of fresh medium containing peptide solution at a final concentration ranging from 0 to 40 μM/well. After 24 h of incubation at 37 °C, the peptide-containing medium was removed, and 100 μL of tetrazolium MTT diluted at 0.5 mg/mL in Dulbecco’s modified Eagle’s medium (DMEM) purchased from Lonza (Basel, Switzerland) without red phenol was added. After 4 h of incubation at 37 °C, the resulting insoluble formazan salts were solubilised in 0.04 M HCl in anhydrous isopropanol and quantified by measuring the absorbance at λ = 570 nm, using an automatic plate reader spectrophotometer (Synergy HTX Multi-Mode Reader-BIOTEK, Winooski, Vermont, United States). Cell survival was expressed as means of the percentage values compared to control. Analyses were performed at the last three times. MTT assays were carried out also to evaluate the dose-dependent effects of GVF27 on the metabolic activity of the bacterial cells [45]. Briefly, bacteria were grown overnight at 37 °C and then diluted to 4×10^8^ CFU/mL in 0.5× MHB. Incubations with increasing concentrations of GVF27 were carried out either for 4 h, in order to test peptide effects on cells attachment, or for 24 h, in order to test peptide effects on biofilm formation. When the effects of peptides on preformed biofilm were evaluated, bacterial biofilm was formed for 24 h at 37 °C and then treated with GVF27. Following the treatment, the bacterial suspension, representing the planktonic cells, was removed, and MTT reagent was added, keeping the microplates at 37 °C. After 3 h, MTT solution was removed, and two washing steps were performed with PBS. Then, DMSO was added to let the dissolution of the formazan crystals that, as above described, were measured at λ = 570 nm.

### 4.7. Anti-Biofilm Activity Assays

Bacteria were grown over-night at 37 °C and then diluted to 4 × 10^8^ CFU/mL in 0.5× MHB. Incubations with increasing concentrations of GVF27 (from 0.078 to 20 μM) were carried out either for 4 h, in order to test peptide effects on cells attachment, or for 24 h, in order to test peptide effects on biofilm formation, as previously described [46]. When the effects of peptides on preformed biofilm were evaluated, bacterial biofilm was formed for 24 h at 37 °C and then treated with GVF27. To carry out the crystal violet assays, bacterial biofilms were gently washed with PBS 1X and then incubated with the dye (0.04%) for 20 min at room temperature. At the end of the incubation and after a further wash with PBS 1X, the dye bound to cells was dissolved in 33% acetic acid. Spectrophotometric analyses were then carried out at a wavelength of 600 nm.

To determine the number of viable planktonic and biofilm-embedded bacteria, following treatment with GVF27, supernatants containing planktonic bacteria were removed and biofilm disrupted with 0.1 % Triton X-100. Both supernatants and disrupted biofilm were diluted and seeded on solid LB-agar plates. Upon incubation for 16 h at 37 °C, the number of colonies forming units was calculated.

Confocal laser scanning microscopy analyses in static conditions were performed, growing bacteria in MHB 0.5× at 37 °C into Thermo Scientific™ Nunc™ Lab-Tek™ Chambered Coverglass systems (Thermo Fisher Scientific, Waltham, MA, USA). To this purpose, bacterial cells from an over-night culture were diluted to about 2 × 10^9^ CFU/mL and seeded into wells of the chambers. After bacterial treatment with GVF27, non-adherent bacteria were removed by gently washing samples with sterile 10 mM sodium phosphate buffer at pH 7.4. The viability of the cells embedded in the biofilm structure was evaluated by sample staining with either LIVE/DEAD^®^ Bacterial Viability kit (Molecular Probes Thermo Fisher Scientific, Waltham, MA, USA) or with FilmTracer™ SYPRO™ Ruby Biofilm Matrix Stain (Invitrogen™), accordingly to the manufacturers’ instructions. The acquisitions were carried out in two different chambers, using for each fluorophore its specific excitation parameter. Biofilm images were captured by using a confocal laser scanning microscope (Zeiss LSM 710, Zeiss, Germany) and a 63X objective oil-immersion system. Biofilm architecture was analysed by using the Zen Lite 2.3 software package. Each experiment was performed in triplicate. All the images were taken under identical conditions.

### 4.8. Minimal Agglutination Concentration (MAC)

*B. cenocepacia* LMG 18863 cells were grown at 37 °C to an OD_600 nm_ of 0.2, centrifuged at 5000 × *g* for 2 min, and resuspended in NaP buffer (pH 7.4) to give an absorbance at 600 nm of 1. An aliquot of 300 µL of the bacterial suspension was treated with increasing peptide concentrations (from 0 to 40 µM) in the Lab-Tek™ Chambered Cover glass systems and incubated at room temperature for 1 h. The aggregation behaviour was observed by staining with wheat germ agglutinin-Oregon Green 488 conjugate (50 µg/mL) by confocal laser scanning microscopy. The agglutinating activity is expressed as the minimum agglutinating concentration (MAC) of the samples tested. Each experiment was performed in triplicate.

### 4.9. Scanning Electron Microscopy

To perform scanning electron microscopy (SEM) analyses, *B. cenocepacia* LMG 18863 biofilm was incubated with increasing concentrations of GVF27 (2.5 to 40 μM) for 24 h at 37 °C. Following incubation, bacterial biofilm was fixed in 2.5% glutaraldehyde. Following over-night incubation, bacterial biofilm was washed three times in distilled water and then dehydrated with a graded ethanol series: 25% ethanol (1 × 10 min), 50% ethanol (1 × 10 min), 75% ethanol (1 × 10 min), 95% ethanol (1 × 10 min), and 100% anhydrous ethanol (3 × 30 min). Bacterial biofilm deposited onto glass substrate was sputter coated with a thin layer of Au-Pd (Sputter Coater Denton Vacuum DeskV) to allow subsequent morphological characterisation using a FEI Nova NanoSEM 450 at an accelerating voltage of 5 kV with Everhart Thornley Detector (ETD) and Through Lens Detector (TLD) at high magnification.

### 4.10. LPS Isolation

*B. cenocepacia* J2315 cells were grown overnight in NB at 37 °C, harvested by centrifugation, and freeze-dried. Afterwards, these cells were then submitted to the petroleum ether–chloroform–phenol (PCP) extraction method; the remaining pellet was further extracted according to the hot water–phenol method. Each phase was dialyzed against distilled water to remove phenol, freeze-dried, and then screened by SDS-PAGE to detect the presence of LPS by using the silver staining procedure [47,48]. LPS was identified in the water phase of the hot water–phenol extraction (yield 77.2 mg/g of cells). This material was further purified through an enzymatic digestion using DNAse, RNAse and proteinase K [49], followed by centrifugation at 6000 rpm for 30 min at 4 °C. The purified LPS was isolated in the pellet (yield 15.0 mg/g of cells).

### 4.11. Isothermal Titration Calorimetry

Isothermal titration calorimetry (ITC) was performed on the Low Volume NanoITC (TA Instruments-Waters LLC, New Castle, DE, USA) to determine interaction between LPSs from *B. cenocepacia* or *E. coli* and GVF27. Briefly, both GVF27 (200 µM) and LPS (0.188 mg/mL, ~12.5 µM) were diluted in 10 mM HEPES, pH 7. The chamber was filled with 164 µL of LPS, and 2 µL of the peptide was titrated into the chamber every 300 s. Experiments were performed at 37 °C and analyzed using the Nano Analyze software (TA instruments-Waters LLC). All the experiments were performed in duplicate, after which calculated binding characteristics of both experiments were averaged.

### 4.12. Fluorescence Displacement Assay

Association of GVF27 with LPS has been determined as described in [50]. Briefly, the synthetic FAM-GVF27 (1.25 μM) was added to increasing concentrations of LPSs extracted from *B. cenocepacia* (from 0 to 150 μg/mL), and the fluorescence was monitored at an excitation wavelength of 495 nm and an emission wavelength of 520 nm in 5 mM ammonium acetate (pH 5.0).

### 4.13. LPS Neutralisation Assay by ELISA

Anti-inflammatory effects of GVF27 were analysed in PMA differentiated and undifferentiated THP-1 cells treated with LPS. Cells were plated into 96-well plates (round-bottomed for undifferentiated suspended cells and flat-bottomed for differentiated adherent cells) at a density of 2 × 10^4^ cells in 100 µL of medium per well. Following incubation with 5 or 20 µM GVF27 and 1µg/mL LPS, the medium was collected to quantify cytokines levels. TNF-α and MCP-1 levels in collected supernatants were determined by using human immunoassay kits (DuoSet ELISA kits, R&D Systems, Minneapolis, MN) according to the manufacturer’s instructions. Samples optical density was measured using an ELISA reader set at 450 nm with a wavelength correction set at 540 nm. All experiments were performed in triplicate.

### 4.14. Statistical Analysis

Data were analysed with GraphPad Prism, version 5.0 software (GraphPad Inc., San Diego, CA, USA) by using Student’s *t*-test. A *p*-value of 0.05 or less was considered statistically significant.

## 5. Conclusions

Obtained findings shed a new light on potential effects of GVF27 against *B. multivorans* and *B. cenocepacia* and clearly suggest that this peptide is adaptable to function as a suitable template to design effective drugs for the treatment of MDR organisms. Considerably, to the best of our knowledge, GVF27 is the only unmodified cryptic human peptide, in terms of sequence and amino acid residues, to exhibit a broad spectrum of bioactivities towards strains belonging to Bcc complex. The global increase in multi-drug-resistant pathogens along with a steady decline in the discovery of new antibiotics underscores the need for new therapies to control infections; the case of GVF27 confirms how immense and practically still unknown is the potentiality of the human proteome as a guiding tool for the design of a new generation of peptide therapeutic agents.

## Figures and Tables

**Table 1:** Minimum inhibitory concentration values (MIC, μM) determined for GVF27, FAM-GVF27, and LL-37 on *B. cenocepacia* LMG 18863 and *B. multivorans* LMG 17582. LL-37 was used as reference human antimicrobial peptide. FAM-GVF27 is 5,6-FAM fluorescently labelled GVF27 used in experiments described in the main text (see page 8 and 10). Ciprofloxacin is the quinolone antibiotic used in synergism experiments described in the main text (see page 11). MIC values were obtained from a minimum of three independent experiments.

		MIC (μM)		
Bcc Strain	GVF27	FAM-GVF27	LL-37	Ciprofloxacin
*Burkholderia cenocepacia* LMG 18863	40	40	5	2.5
*Burkholderia multivorans* LMG 17582	10	10	10	1.2

**Table 2:** Sequences of GVF27 and LL-37 peptides.

Peptide	Sequence
GVF27	^1^ GVFYPWRFRLLCLLRRWLPRPRAWFIR^27^
LL-37	^1^ LLGDFFRKSKEKIGKEFKRIVQRIKDFLRNLVPRTES^37^

## Data Availability

Data is contained within the article or Supplementary Materials.

## Supplementary Materials

The following supporting information can be downloaded at: https://www.mdpi.com/article/10.3390/ph15020260/s1, Video S1: Time course of labeled GVF27; Video S2: Time course of labeled GVF27 and SYPRO Ruby.

## References

[1] Prestinaci F., Pezzotti P., Pantosti A. (2015). Antimicrobial resistance: A global multifaceted phenomenon. Pathog. Glob. Health.

[2] Hutchings M., Truman A., Wilkinson B. (2019). Antibiotics: Past, present and future. Curr. Opin. Microbiol..

[3] Payne D.J., Miller L.F., Findlay D., Anderson J., Marks L. (2015). Time for a change: Addressing R&D and commercialization challenges for antibacterials. Philos. Trans. R. Soc. B Biol. Sci..

[4] Hancock R.E.W., Haney E.F., Gill E.E. (2016). The immunology of host defence peptides: Beyond antimicrobial activity. Nat. Rev. Immunol..

[5] Fjell C.D., Hiss J.A., Hancock R.E.W., Schneider G. (2011). Designing antimicrobial peptides: Form follows function. Nat. Rev. Drug Discov..

[6] Hemshekhar M., Anaparti V., Mookherjee N. (2016). Functions of cationic host defense peptides in immunity. Pharmaceuticals.

[7] Mangoni M.L., Mcdermott A.M., Zasloff M. (2016). Antimicrobial peptides and wound healing: Biological and therapeutic considerations. Exp. Dermatol..

[8] Park Y.J., Lee S.K., Jung Y.S., Lee M., Lee H.Y., Kim S.D., Park J.S., Koo J.H., Hwang J.S., Bae Y.S. (2016). Promotion of formyl peptide receptor 1-mediated neutrophil chemotactic migration by antimicrobial peptides isolated from the centipede *Scolopendra subspinipes mutilans*. BMB Rep..

[9] Veldhuizen E.J.A., Schneider V.A.F., Agustiandari H., Van Dijk A., Tjeerdsma-van Bokhoven J.L.M., Bikker F.J., Haagsman H.P. (2014). Antimicrobial and immunomodulatory activities of PR-39 derived peptides. PLoS ONE.

[10] Niyonsaba F., Ushio H., Nakano N., Ng W., Sayama K., Hashimoto K., Nagaoka I., Okumura K., Ogawa H. (2007). Antimicrobial peptides human β-defensins stimulate epidermal keratinocyte migration, proliferation and production of proinflammatory cytokines and chemokines. J. Investig. Dermatol..

[11] Oppenheim J.J., Biragyn A., Kwak L.W., Yang D. (2003). Roles of antimicrobial peptides such as defensins in innate and adaptive immunity. Ann. Rheum. Dis..

[12] Lewies A., Du Plessis L.H., Wentzel J.F. (2018). Antimicrobial Peptides: The Achilles’ Heel of Antibiotic Resistance? Probiotics Antimicrob. Proteins.

[13] Sun E., Belanger C.R., Haney E.F., Hancock R.E.W. (2018). Host defense (antimicrobial) peptides. Peptide Applications in Biomedicine, Biotechnology and Bioengineering.

[14] Drevinek P., Mahenthiralingam E. (2010). *Burkholderia cenocepacia* in cystic fibrosis: Epidemiology and molecular mechanisms of virulence. Clin. Microbiol. Infect..

[15] Mahenthiralingam E., Urban T.A., Goldberg J.B. (2005). The multifarious, multireplicon *Burkholderia cepacia* complex. Nat. Rev. Genet..

[16] Melo Coutinho H. (2007). *Burkholderia cepacia* complex: Virulence characteristics, importance and relationship with cystic fibrosis. Indian J. Med. Sci..

[17] Rhodes K.A., Schweizer H.P. (2016). Antibiotic resistance in *Burkholderia* species. Drug Resist. Updat..

[18] Podnecky N.L., Rhodes K.A., Schweizer H.P. (2015). Efflux pump-mediated drug resistance in *Burkholderia*. Front. Microbiol..

[19] Shommu N.S., Vogel H.J., Storey D.G. (2015). Potential of metabolomics to reveal *Burkholderia cepacia* complex pathogenesis and antibiotic resistance. Front. Microbiol..

[20] Bellich B., Jou I.A., Buriola C., Ravenscroft N., Brady J.W., Fazli M., Tolker-Nielsen T., Rizzo R., Cescutti P. (2021). The biofilm of *Burkholderia cenocepacia* H111 contains an exopolysaccharide composed of L-rhamnose and L-mannose: Structural characterization and molecular modelling. Carbohydr. Res..

[21] Terán L.C., Distefano M., Bellich B., Petrosino S., Bertoncin P., Cescutti P., Sblattero D. (2020). Proteomic studies of the biofilm matrix including outer membrane vesicles of *Burkholderia multivorans* c1576, a strain of clinical importance for cystic fibrosis. Microorganisms.

[22] McClean S., Callaghan M. (2009). *Burkholderia cepacia* complex: Epithelial cell-pathogen confrontations and potential for therapeutic intervention. J. Med. Microbiol..

[23] Kooi C., Subsin B., Chen R., Pohorelic B., Sokol P.A. (2006). *Burkholderia cenocepacia* ZmpB is a broad-specificity zinc metalloprotease involved in virulence. Infect. Immun..

[24] Mullen T., Markey K., Murphy P., McClean S., Callaghan M. (2007). Role of lipase in *Burkholderia cepacia* complex (Bcc) invasion of lung epithelial cells. Eur. J. Clin. Microbiol. Infect. Dis..

[25] Ganesan S., Sajjan U.S. (2012). Host evasion by *Burkholderia cenocepacia*. Front. Cell. Infect. Microbiol..

[26] Shaw D., Poxton I.R., Govan J.R.W. (1995). Biological activity of *Burkholderia* (*Pseudomonas*) *cepacia* lipopolysaccharide. FEMS Immunol. Med. Microbiol..

[27] De Soyza A., Silipo A., Lanzetta R., Govan R.J., Molinaro A. (2008). Chemical and biological features of *Burkholderia cepacia* complex lipopolysaccharides. Innate Immun..

[28] Hollaus R., Ittig S., Hofinger A., Haegman M., Beyaert R., Kosma P., Zamyatina A. (2015). Chemical synthesis of burkholderia lipid a modified with glycosyl phosphodiester-linked 4-amino-4-deoxy-β-L-arabinose and its immunomodulatory potential. Chem. A Eur. J..

[29] Pizzo E., Cafaro V., Di Donato A., Notomista E. (2018). Cryptic antimicrobial peptides: Identification methods and current knowledge of their immunomodulatory properties. Curr. Pharm. Des..

[30] Bosso A., Di Maro A., Cafaro V., Di Donato A., Notomista E., Pizzo E. (2020). Enzymes as a Reservoir of Host Defence Peptides. Curr. Top. Med. Chem..

[31] Pane K., Durante L., Crescenzi O., Cafaro V., Pizzo E., Varcamonti M., Zanfardino A., Izzo V., Di Donato A., Notomista E. (2017). Antimicrobial potency of cationic antimicrobial peptides can be predicted from their amino acid composition: Application to the detection of “cryptic” antimicrobial peptides. J. Theor. Biol..

[32] Bosso A., Pirone L., Gaglione R., Pane K., Del Gatto A., Zaccaro L., Di Gaetano S., Diana D., Fattorusso R., Pedone E. (2017). A new cryptic host defense peptide identified in human 11-hydroxysteroid dehydrogenase-1 β-like: From in silico identification to experimental evidence. Biochim. Biophys. Acta Gen. Subj..

[33] Yakandawala N., Gawande P.V., LoVetri K., Cardona S.T., Romeo T., Nitz M., Madhyastha S. (2011). Characterization of the poly-β-1,6-N-acetylglucosamine polysaccharide component of *Burkholderia* biofilms. Appl. Environ. Microbiol..

[34] Di Lorenzo F., Kubik Ł., Oblak A., Lorè N.I., Cigana C., Lanzetta R., Parrilli M., Hamad M.A., De Soyza A., Silipo A. (2015). Activation of Human Toll-like receptor 4 (TLR4)·myeloid differentiation factor 2 (MD-2) by hypoacylated lipopolysaccharide from a clinical isolate of *Burkholderia cenocepacia*. J. Biol. Chem..

[35] Speert D.P., Henry D., Vandamme P., Corey M., Mahenthiralingam E. (2002). Epidemiology of *Burkholderia cepacia* complex in patients with cystic fibrosis, Canada. Emerg. Infect. Dis..

[36] Loutet S.A., Valvano M.A. (2011). Extreme Antimicrobial Peptide and Polymyxin B Resistance in the Genus *Burkholderia*. Front. Microbiol..

[37] Loutet S.A., Valvano M.A. (2010). A decade of Burkholderia cenocepacia virulence determinant research. Infect. Immun..

[38] Van Acker H., Sass A., Bazzini S., De Roy K., Udine C., Messiaen T., Riccardi G., Boon N., Nelis H.J., Mahenthiralingam E. (2013). Biofilm-Grown *Burkholderia cepacia* Complex Cells Survive Antibiotic Treatment by Avoiding Production of Reactive Oxygen Species. PLoS ONE.

[39] Srikantha T., Daniels K.J., Pujol C., Kim E., Soll D.R. (2013). Identification of genes upregulated by the transcription factor bcr1 that are involved in impermeability, impenetrability, and drug resistance of *Candida albicans* a/α biofilms. Eukaryot. Cell.

[40] Hoffman L.R., D’Argenio D.A., MacCoss M.J., Zhang Z., Jones R.A., Miller S.I. (2005). Aminoglycoside antibiotics induce bacterial biofilm formation. Nature.

[41] Linares J.F., Gustafsson I., Baquero F., Martinez J.L. (2006). Antibiotics as intermicrobiol signaling agents instead of weapons. Proc. Natl. Acad. Sci. USA.

[42] Sharafutdinov I.S., Ozhegov G.D., Sabirova A.E., Novikova V.V., Lisovskaya S.A., Khabibrakhmanova A.M., Kurbangalieva A.R., Bogachev M.I., Kayumov A.R. (2020). Increasing Susceptibility of Drug-Resistant *Candida albicans* to Fluconazole and Terbinafine by 2(5H)-Furanone Derivative. Molecules.

[43] Cunha M.V., Leitão J.H., Mahenthiralingam E., Vandamme P., Lito L., Barreto C., Salgado M.J., Sá-Correia I. (2003). Molecular analysis of *Burkholderia cepacia* complex isolates from a Portuguese cystic fibrosis center: A 7-year study. J. Clin. Microbiol..

[44] Wiegand I., Hilpert K., Hancock R.E.W. (2008). Agar and broth dilution methods to determine the minimal inhibitory concentration (MIC) of antimicrobial substances. Nat. Protoc..

[45] Fratianni F., d’Acierno A., Ombra M.N., Amato G., De Feo V., Ayala-Zavala J.F., Coppola R., Nazzaro F. (2021). Fatty Acid Composition, Antioxidant, and in vitro Anti-inflammatory Activity of Five Cold-Pressed *Prunus* Seed Oils, and Their Anti-biofilm Effect Against Pathogenic Bacteria. Front. Nutr..

[46] Gaglione R., Dell’Olmo E., Bosso A., Chino M., Pane K., Ascione F., Itri F., Caserta S., Amoresano A., Lombardi A. (2017). Novel human bioactive peptides identified in Apolipoprotein B: Evaluation of their therapeutic potential. Biochem. Pharmacol..

[47] Kittelberger R., Hilbink F. (1993). Sensitive silver-staining detection of bacterial lipopolysaccharides in polyacrylamide gels. J. Biochem. Biophys. Methods.

[48] Tsai C.M., Frasch C.E. (1982). A sensitive silver stain for detecting lipopolysaccharides in polyacrylamide gels. Anal. Biochem..

[49] De Castro C., Parrilli M., Holst O., Molinaro A. (2010). Microbe-associated molecular patterns in innate immunity. Extraction and chemical analysis of gram-negative bacterial lipopolysaccharides. Methods Enzymol..

[50] Mohanram H., Bhattacharjya S. (2014). Resurrecting inactive antimicrobial peptides from the lipopolysaccharide trap. Antimicrob. Agents Chemother..

